# Local and global pyrogeographic evidence that indigenous fire management creates pyrodiversity

**DOI:** 10.1002/ece3.1494

**Published:** 2015-04-14

**Authors:** Clay Trauernicht, Barry W Brook, Brett P Murphy, Grant J Williamson, David M J S Bowman

**Affiliations:** 1 Department of Natural Resources and Environmental Management, College of Tropical Agriculture and Human Resources, University of Hawai’i at MānoaHonolulu, Hawai’i, 96822; 2 School of Plant Science, University of TasmaniaHobart, Tasmania, 7001, Australia; 3 Environment Institute and School of Earth and Environmental Sciences, University of AdelaideAdelaide, South Australia, 5005, Australia; 4 NERP Environmental Decisions Hub, School of Botany, The University of MelbourneMelbourne, Victoria, 3010, Australia

**Keywords:** Coupled human and natural systems, fire ecology, heterogeneity, indigenous burning, landscape burning, patch mosaic burning, pyrodiversity

## Abstract

Despite the challenges wildland fire poses to contemporary resource management, many fire-prone ecosystems have adapted over centuries to millennia to intentional landscape burning by people to maintain resources. We combine fieldwork, modeling, and a literature survey to examine the extent and mechanism by which anthropogenic burning alters the spatial grain of habitat mosaics in fire-prone ecosystems. We survey the distribution of *Callitris intratropica*, a conifer requiring long fire-free intervals for establishment, as an indicator of long-unburned habitat availability under Aboriginal burning in the savannas of Arnhem Land. We then use cellular automata to simulate the effects of burning identical proportions of the landscape under different fire sizes on the emergent patterns of habitat heterogeneity. Finally, we examine the global extent of intentional burning and diversity of objectives using the scientific literature. The current distribution of *Callitris* across multiple field sites suggested long-unburnt patches are common and occur at fine scales (<0.5 ha), while modeling revealed smaller, patchy disturbances maximize patch age diversity, creating a favorable habitat matrix for *Callitris*. The literature search provided evidence for intentional landscape burning across multiple ecosystems on six continents, with the number of identified objectives ranging from two to thirteen per study. The fieldwork and modeling results imply that the occurrence of long-unburnt habitat in fire-prone ecosystems may be an emergent property of patch scaling under fire regimes dominated by smaller fires. These findings provide a model for understanding how anthropogenic burning alters spatial and temporal aspects of habitat heterogeneity, which, as the literature survey strongly suggests, warrant consideration across a diversity of geographies and cultures. Our results clarify how traditional fire management shapes fire-prone ecosystems, which despite diverse objectives, has allowed human societies to cope with fire as a recurrent disturbance.

## Introduction

Wildfire poses enormous challenges for contemporary land management and resource protection. Policy discourse has shifted from outright fire suppression to building fire-adapted and fire-resilient landscapes and communities. A key to achieving sustainable coexistence with fire is in better understanding the ancient nexus between humans and flammable landscapes. The genus *Homo* likely began manipulating fire c. 1 million years ago (Pausas and Keeley 2009), and evidence indicates burning by modern humans has altered vegetation and other resources across large spatial scales. Indeed, recent departures from traditional cultural use and perceptions of fire are associated with major shifts in ecological composition, ranging from local-scale shrub encroachment and forest degradation to regional- and continental-scale changes in vegetation (Stewart 1951; Bowman et al. 2001; Burrows et al. 2006; Nowacki and Abrams 2008; Bilbao et al. 2010; Pellatt and Gedalof 2014).

Intentional landscape burning is a powerful tool with which humans have managed plant and animal productivity and availability for millennia on most continents (Stewart 1951; Lewis and Ferguson 1988; Pyne 1997; Kimmerer and Lake 2001; Bowman et al. 2011; Huffman 2013). The integration of anthropogenic burning with ecological theory is, however, complicated by the diversity of historical, economic, and cultural contexts surrounding fire management (Murphy et al. 2007; Fowler 2013). Consequently, the nature and scale of ecological outcomes wrought by ‘fire-stick farming’ (Jones 1969), or the manipulation of resources via fire, remains debated. By some accounts, anthropogenic fire has manufactured landscape diversity (Pyne 1997; Boyd 1999; Gammage 2011), whereas others suggest limited human influence relative to natural ignitions (Vale 2002).

Human–fire interactions are one facet of the complex relationship between fire disturbance and ecosystem composition. Fire regimes—defined by the intensity, frequency, extent, and spatial patterns of fire across landscapes—are driven by many biotic, abiotic, and climatic interactions (Bond and Keeley 2005; Archibald et al. 2009). The spatial and temporal dynamics of landscape fire results in a ‘patch mosaic’ of successional habitat described as ‘pyrodiversity’ (Martin and Sapsis 1992). Despite understanding the biophysical drivers of fire, the invisibility of historical burning patterns and its legacy effects on vegetation make pyrodiversity inherently difficult to study (Bradstock et al. 2005). Thus, the influence of humans on past fire ecology is difficult to assess.

Research suggests that intentional landscape burning influences pyrodiversity by altering ignition seasonality and frequency beyond the natural range. Indigenous burning typically occurs under cooler, moister conditions—such as early dry season in tropical savannas of Africa, Australia, and South America (Russell-Smith et al. 1997; Laris 2002; Bilbao et al. 2010) or spring and autumn in temperate regions (Kimmerer and Lake 2001; Macdougall 2004) —than when lightning ignitions occur (e.g., late dry season and summer). These practices may also provide ignition sources in environments that are not subject to lightning strikes. Although these practices may increase ignition frequency, on average they result in smaller, less intense fires that, like contemporary prescribed burning, reduce the physical threat of wildfire and increase habitat heterogeneity (Laris 2002; Bowman et al. 2004; Burrows et al. 2006; Bliege Bird et al. 2008). Despite strong anthropological evidence (Jones 1969; Lewis and Ferguson 1988; Fowler 2013), ecologists remain equivocal on the link between pyrodiversity and plant and animal abundance and diversity (e.g., Parr and Andersen 2006).

Many insights into the interconnectedness of people, fire, and resources come from research among Aboriginal communities in the tropical savannas of Arnhem Land, northern Australia. The region ranks among the world’s most fire prone (i.e., 1–3 year fire-return intervals) and contains some of the oldest continuously managed cultural landscapes on Earth (Yibarbuk et al. 2001). Disease, displacement, and economic development have led to the cessation of Aboriginal management across much of northern Australia within the past century. Thus, a fire regime of patchy, low-intensity fires initiated early in the dry season by a widely dispersed human population has switched to a ‘modern wilderness’ (Bowman et al. 2001), dominated by large (i.e., hundreds of km^2^), high-intensity fires set by lightning in the late dry season (Yates et al. 2008). This change is implicated in declines of multiple taxa, most notably small mammals (Woinarski et al. 2010). Yet arguably the most evident ecological consequence is widespread mortality in one of the savanna’s few noneucalypt overstory trees, the fire-sensitive conifer *Callitris intratropica* R.T. Baker and H.G. Smith (Bowman et al. 2001).

**Figure 1:**
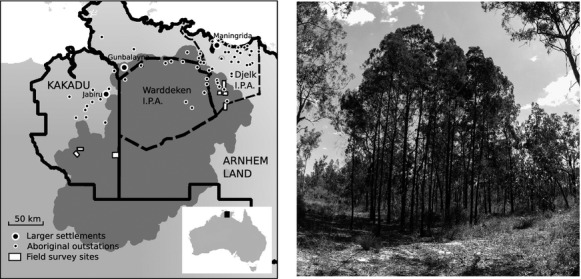
(A) shows field sites (white boxes) where the fire-sensitive conifer, *Callitris intratropica,* was surveyed in Arnhem Land and Kakadu National Park, while (B) illustrates *Callitris* grove formation, in which closed-canopy patches suppress graminoid fuels, exclude low-intensity savanna fires, and maintain small-scale (i.e., <0.5 ha) fire refugia for conspecific recruitment and a distinct shrub community (Trauernicht et al. 2013).

Unlike eucalypts, which resprout prolifically after burning, *Callitris* is vulnerable to fire. It is an obligate-seeder, meaning regeneration from seed is required for the species to persist at a site. Individuals are typically killed by intense fires, and juvenile trees require up to 10 years before they can survive even low-intensity fires (Russell-Smith 2006). Yet despite its sensitivity to fire, *Callitris* remains common across much of the fire-prone savanna vegetation in Arnhem Land. The persistence of *Callitris* has been linked to spatial clumping of the tree into small groves (e.g., <0.5 ha; Fig.1). Closed-canopy *Callitris* groves suppress graminoid fuels, exclude low-intensity fires that approach from the surrounding savanna matrix, and provide refuge for both conspecific recruitment and a distinct community of fire-sensitive shrubs (Trauernicht et al. 2012). High-intensity fires scorch *Callitris* canopies and open grove understories, effectively switching flammability and composition to savanna conditions, often despite the survival of larger *Callitris* individuals. The prevalence of fire-damaged, open-canopy *Callitris* groves in the landscape therefore indicates a predominance of high-intensity fires and lower plant diversity (Trauernicht et al. 2013).

A prevailing hypothesis is that Aboriginal burning allowed *Callitris* to ‘invade’ open savanna vegetation from topographically fire-protected sites by increasing pyrodiversity and creating favorable habitat. Extant old adult trees were recruited 100–200 years ago in a landscape that was then extensively managed by Aboriginal burning (Prior et al. 2011); the species remains abundant in areas where these practices continue (Yibarbuk et al. 2001; Trauernicht et al. 2013). The persistence of *Callitris* groves depends on low-intensity fires; however, the ‘recruitment’ of new groves—occasionally observed in open savanna as clumps of seedling and sapling cohorts—clearly requires long fire-free intervals (i.e., >10 years; Russell-Smith 2006). The available data demonstrate that Aboriginal burning is patchy (Bowman et al. 2004; Vigilante et al. 2004; Burrows et al. 2006; Bliege Bird et al. 2008) and designed to manipulate habitat for a wide variety of food resources (Russell-Smith et al. 1997; Murphy and Bowman 2007), but does not appear designed explicitly to favor *Callitris* (J. Rostron, pers. comm.).

Analyses of fire perimeters also show that increasing the prevalence of low-intensity, early dry season fires through management in tropical savannas does not affect the percentage of the landscape burnt annually (Gill et al. 2000; Van Wilgen et al. 2004). In other words, this indicates that the average fire frequency remains the same whether the landscape burns by many small fires or fewer larger fires. Thus, exactly how Aboriginal burning creates the patches of long-unburnt (≥10 years) habitat required for *Callitris* establishment remains a puzzle. In this context, we sought to better understand the ecological outcomes of anthropogenic burning using the spatial distribution of *Callitris* groves in Arnhem Land and Kakadu National Park to examine fine-scale patterns in the availability of long-unburnt habitat. We then deployed a simple cellular automaton simulation model to explore how altering fire size, and therefore the spatial grain of fire occurrence, affects both spatial and temporal aspects of pyrodiversity.

Despite the complexity of factors influencing fire behavior and effects, the relative simplicity of our study system and modeling approach provide a unique opportunity to examine a fundamental question of patch mosaic burning: how does human mediation of fire size, irrespective of area burned, alter habitat complexity? We argue that this question is critical to understanding traditional fire management as a coupled human and natural system at the global scale, both in terms of how intentional burning has shaped baseline ecological patterns and how the outcomes of landscape burning give rise to fire-resilient communities and landscapes. We therefore turned to the available literature to examine the global extent of landscape burning as a cultural practice and consider the relationship between the explicit objectives of fire management and its potential ecological outcomes as indicated by the fire disturbance simulation.

## Methods

We surveyed the density and size of *Callitris* groves across expanses of savanna vegetation in three areas of Kakadu National Park (KNP) and three Aboriginal estates in central Arnhem Land (CAL; see Trauernicht et al. 2013 for site descriptions). Areas of *Callitris* occurrence were identified by discussions with Park Rangers and Aboriginal landowners, with each survey area topographically delineated as an open, level tract, or ‘basin’ (i.e., tens of km^2^), of *Eucalyptus tetrodonta*/*E. miniata* savanna. We conducted two field traverses in each area for a total of 12 transects ranging in length from 1.5 to 5 km (33.7 km total; transect lengths differed due to topographic features) and counted all groves encountered within 50 m of transect center. Due to the time constraints inherent in accessing transects in remote areas, grove size (area) was measured for a random subset of groves.

We constructed a simple cellular automaton (CA), or lattice model, driven as a stochastic simulation, to examine the effects of fire size on the spatial and temporal heterogeneity of different ‘aged’ cells across a two-dimensional landscape. A spatially explicit CA is useful for examining complex, emergent patterns from simple rule sets and have been used extensively to model the ecological effects of fire (Perry and Enright 2006). Whereas a CA typically models fire spread based on multiple parameters, we employed Green’s (1989) simplified and more tractable approach of uniform fuel type, fuel replacement between fires, randomly located ignitions, and constant fire size, to simulate fire regimes that burned the same annual total area under different combinations of fire size and number. These characteristics were chosen to represent the relative uniformity of grassy, surface fuels of northern Australian savannas and, more importantly, isolate the effect of reducing fire size, a demonstrated outcome of intentional burning across multiple ecosystems (Mbow et al. 2000; Laris 2002; Bowman et al. 2004; Bliege Bird et al. 2008; Bilbao et al. 2010). Thus, for each time step (i.e., year), the model randomly placed squares of a specified size and number, representing burned areas, across a 150 × 150 pixel landscape. Each parameterization of given fire size and number was run for 150 years (based on preliminary assessments of time required for landscape patterns to equilibrate) and replicated 100 times.

Assuming the total automaton extent (i.e., 22,500 cells) represented a 3 × 3 km landscape, we ran fire-size parameterizations of 1 ha (i.e., 5 × 5 cells), 2 ha, 5 ha, 10 ha, 20 ha, 30 ha, 40 ha, 50 ha, and 75 ha and adjusted fire number parameterizations so that each ‘treatment’ burned, on average adjusting for fire footprint overlap, 40% of the landscape—the mean value recorded for northern Australian savannas from 1980–1995 (Gill et al. 2000). The simulation operated as a Markov chain, with the fire history of a given cell having no effect on subsequent time steps, allowing for annual fire-return times (more typical of frequently burnt biomes like mesic savannas). At the end of each model run, we sampled the count of fires and time since the last fire (TSF) for each cell within a 50 × 50 cell ‘plot’ at the landscape center (to avoid boundary effects). The count of fires per cell was used to measure fire frequency, and contiguous cells with the same TSF values were interpreted as discrete habitat patches, from which we described habitat composition based on the count, size, and age of discrete habitat patches within the plot. We then used boxplots to compare the distributions of the following response variables across each fire-size parameterization: median and maximum fire-return time; median and maximum patch age; Shannon’s index of patch age diversity; median patch size (plotted on a natural log scale); and spatial heterogeneity (an adjacency index of the degree to which the value of a central cell differs from the values of neighboring cells; larger adjacency values indicate greater heterogeneity). We used this same approach to examine the density of all discrete habitat patches, the density of patches >5 years old, and the density of patches >10 years old based on the time required for *Callitris* establishment (10 years; Russell-Smith 2006).

To examine the global extent of indigenous landscape burning and the diversity of its application for resource management, we used Google Scholar and Web of Science with the search terms ‘fire’ and ‘burning’ each combined with the terms ‘traditional’, ‘anthropogenic’, ‘cultural’, ‘indigenous’, ‘aboriginal’, ‘First Nation’, and ‘Native American’ to identify relevant literature. We used literature cited by papers from our initial search for a more complete bibliography of research articles, book chapters, and theses on intentional landscape burning among indigenous and, for several cases, rural communities not identified as indigenous (see Appendix S1 in Supporting Information). For each study, we classified findings as based on direct ethnography, historical accounts, or descriptive accounts without reference to sources and categorized and tallied all purposes/objectives of fire use mentioned in each study.

## Results

We recorded 182 *Callitris* groves across our field transects, resulting in a mean density of 1.1 groves ha^−1^ ±0.1 (SE). The 134 groves we measured were consistently small, ranging from 0.005 to 0.34 ha with a median size of 0.025 ha.

These data provided context in terms of indicating the ‘grain’ at which long-unburnt habitat is available in this ecosystem, whereas we turned to the CA simulations to provide insight into the mechanisms by which habitat mosaics are shaped by fire disturbance.

**Figure 2:**
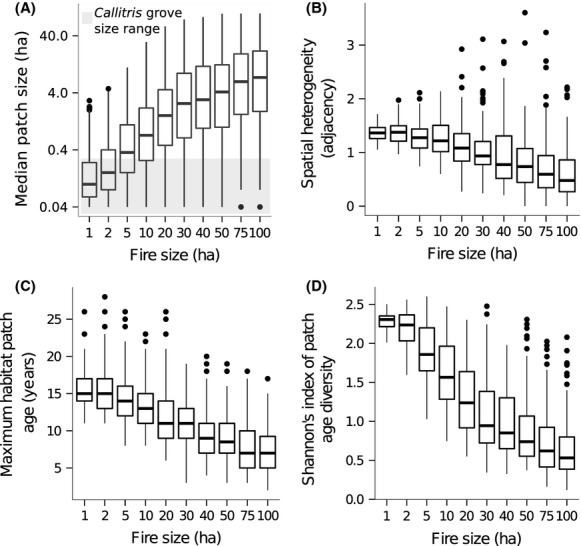
The results from the cellular automaton simulation of fire disturbance, illustrating the effect of fire size on emergent habitat configurations sampled from a 100 ha (50 × 50 cell) plot at the center of a 900 ha (150 × 150 cell) landscape: (A) median habitat patch size (log scale); (B) spatial heterogeneity; (C) maximum habitat patch age; and (D) Shannon’s index of patch age diversity. Horizontal bars represent median values, boxes indicate the first and third quartiles, whiskers show the highest and lowest values within 1.5*IQR (the interquartile range), and points represent data lying outside this range.

**Figure 3:**
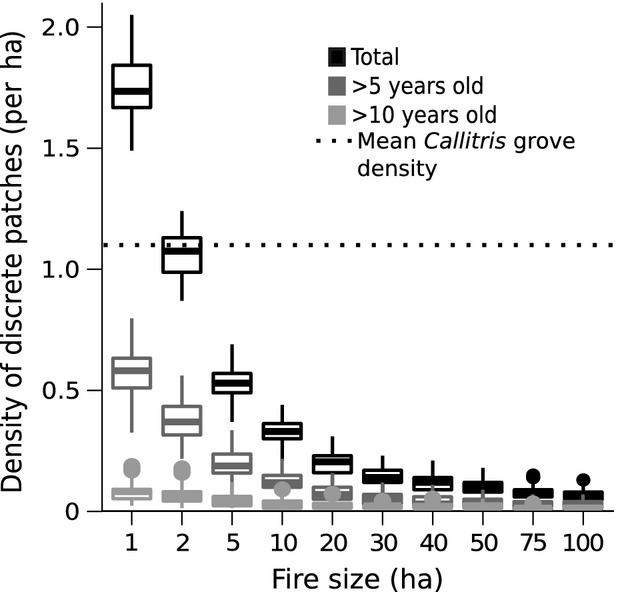
Results of fire simulations under different prevailing fire sizes for the total count of (i) discrete habitat patches (black), (ii) the count of habitat patches >5 years old (dark gray), and (iii) the count of habitat patches >10 years old (light gray), as sampled from a 100 ha plot at the center of a 900 ha landscape. Horizontal bars represent median values, boxes indicate the first and third quartiles, whiskers show the highest and lowest values within 1.5*IQR (the interquartile range), and points represent data lying outside this range.

The most intuitive results from the disturbance model were that smaller fire size increased spatial heterogeneity and decreased median habitat patch size (Fig.2A and B). Although median fire-return time and patch age were consistent across fire-size ‘treatments’ (reflecting the fixed 40% annual area burnt), smaller fires generated larger maximum values for these metrics (Fig.2C). Smaller fires also increased Shannon index of patch age diversity (Fig.2D) and the counts of older (i.e., long-unburnt) habitat patches (Fig.3).

**Figure 4:**
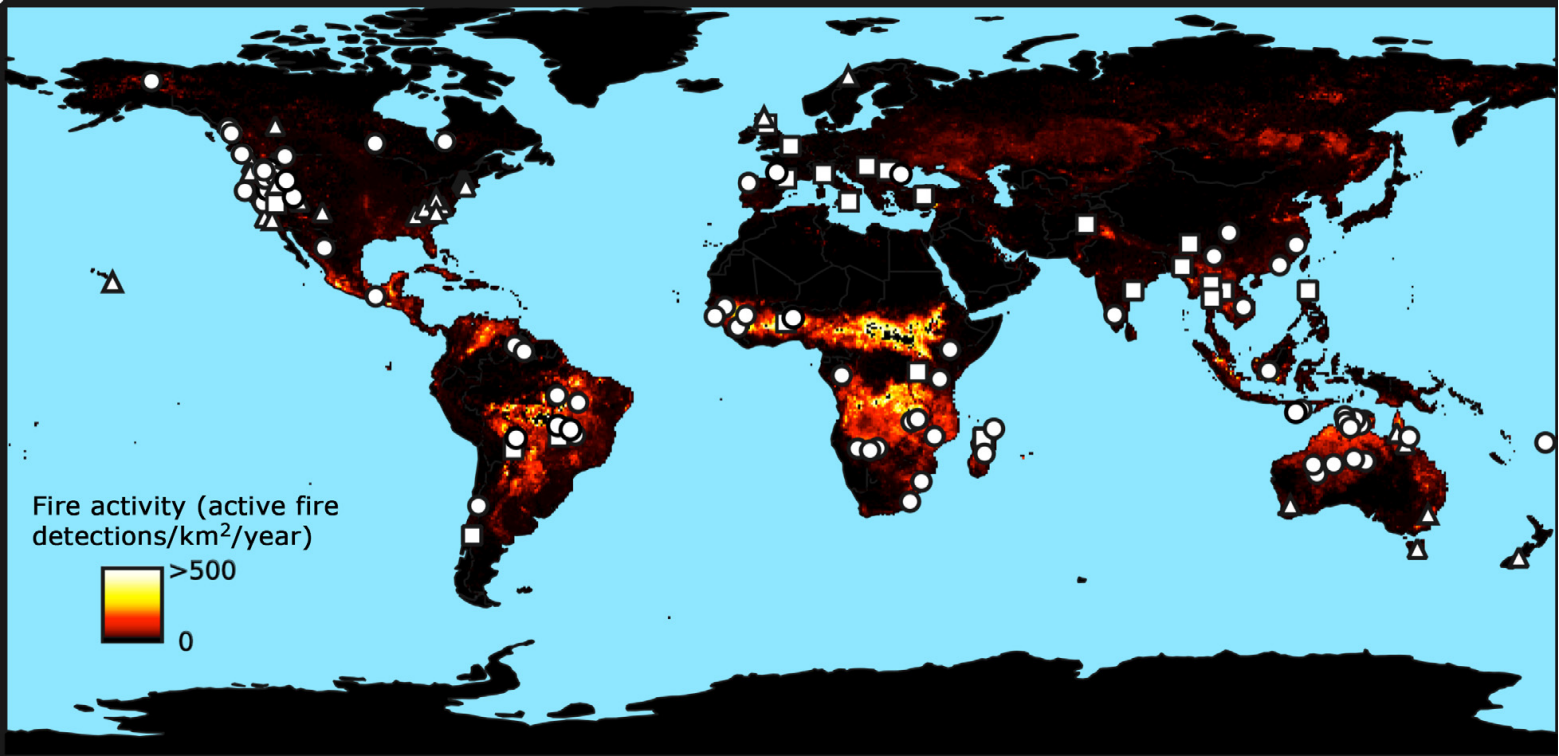
The global distribution of indigenous and rural landscape burning covered in the literature (see Appendix S1). Circles indicate ethnographic studies (*N* = 74), triangles research using historical accounts (*N* = 29), and squares studies that provide descriptive accounts (*N* = 22) without reference to specific sources or data. Color coding illustrates ecosystem flammability using the mean annual density of active fire detections from MODIS satellite data between 2001 and 2006 (Giglio et al. 2006).

**Figure 5:**
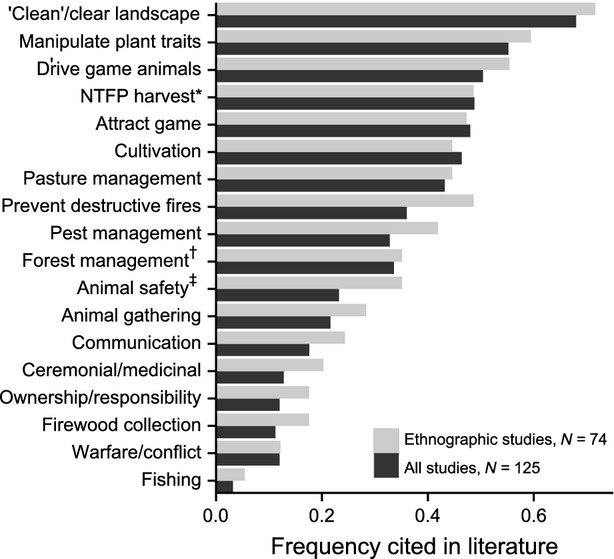
The frequency of objectives of intentional burning cited within research on indigenous and rural fire management practices, presented for ethnographic studies (i.e., interviews with practitioners; *N* = 74) and all surveyed literature (*N* = 125), including ethnographic, historical (*N* = 29), and descriptive accounts (i.e., without specific reference to sources; *N* = 22). See Appendix S1 for references. *Nontimber Forest Products; ^†^Refers explicitly to burning within forests, for example to open the understory or promote recruitment; and ^‡^Refers to reducing risk to lives and livelihoods, such as from predators and venomous snakes.

**Figure 6:**
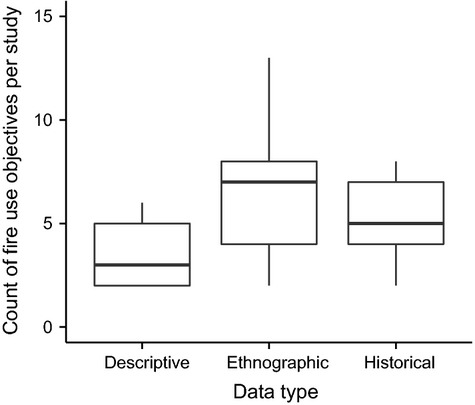
Boxplots illustrating the count of objectives for intentional burning given per study from research on indigenous and rural fire management practices based on descriptive accounts (i.e., without specific reference to sources; *N* = 22), ethnographic studies (i.e., interviews with practitioners; *N* = 74), and historical accounts (derived from archival material like explorers’ journals *N* = 29). See Appendix S1 for references. Horizontal bars represent median values, boxes indicate the first and third quartiles, and whiskers show the highest and lowest values within 1.5*IQR (the interquartile range).

We examined 125 papers (Table in Appendix S1) that explored the relationship between intentional landscape burning and resource availability across a wide range of temperate and tropical biomes (Fig.4). We categorized the purposes and uses of fire across 18 general management objectives (Fig.5). All studies cited multiple management applications of intentional burning, with the number of uses per study highest among the ethnographic literature (Fig.6). ‘Cleaning’ landscapes, defined as using fire to clear and maintain open vegetation, was mentioned most frequently (68% of all studies), followed by manipulating wild plant traits and driving game animals (55% and 50% of all studies, respectively). Intentional burning to protect resources by preventing high-intensity, destructive fires was more frequently cited in ethnographic studies (49%) than across all studies (36%; Fig.5).

## Discussion

Our results support the idea that the composition of flammable ecosystems is not simply a function of how much burns—as conveyed by mean fire frequency and total area burnt—but also the spatial pattern of burning (Laris 2002; Vigilante et al. 2004; Burrows et al. 2006). The effects of fire size on median habitat patch size and habitat heterogeneity in our CA simulation (Fig.2A and B) support the fairly intuitive hypothesis that small fires result in patchier landscapes. Less intuitive, however, were the simulated effects of fire size on the temporal heterogeneity of the landscape. Smaller fires increased maximum habitat patch age, maximum fire-return time, and Shannon’s index of overall patch age diversity (Fig.2C and D). This indicates that disturbance regimes characterized by many small fires increase the occurrence of habitat patches that are older and burned less frequently when compared to fewer, larger fires burning the same proportion of the landscape.

Given the dependence of *Callitris* on fire-free intervals for establishment (e.g., >10 years; Russell-Smith 2006), the spatial distribution of *Callitris* groves suggested long-unburnt habitat patches are, or at least were once, common and occur at fine scales (<0.5 ha). Assuming our simulation represents a 3 × 3 km landscape, the range of median patch sizes under smaller fires (.04–2.9 ha; Fig.2A) approached the range of *Callitris* grove areas measured in the field (0.005–0.34 ha). The count of habitat patches >5 and >10 years old (Fig.3) under the smallest fire size (1 ha), however, was still less than the densities of *Callitris* groves encountered in the field (1.1 groves ha^−1^ ±0.1). But the count of older (unburnt) habitat patches in the model results need not be exactly concordant with *Callitris* grove density to provide insight into the phenomenon of grove establishment. *Callitris* is a long-lived tree (100–200 years), and it is highly unlikely that extant groves established contemporaneously. Therefore, current grove densities provide an overestimate of the expected density of long-unburnt habitat patches under a patch mosaic burning regime. More importantly, even without exact convergence between modeling and field observations, the simulation results strongly suggest that the likelihood of long-unburnt habitat patches occurring in landscapes dominated by large fires is extremely low (e.g., Fig.3).

Obviously, the fixed fire size and lack of ‘memory’ in our simulation provide a highly simplified model of real-world disturbance dynamics. For instance, large fires will likely occur under any management regime and may create habitat heterogeneity in some systems (Knapp and Keeley 2006; Bradstock 2009). Questions remain regarding the degree to which anthropogenic fires actually reduce the occurrence of large fires over longer time scales and how quickly patch mosaics re-establish after large disturbances (Bradstock et al. 2005; Price et al. 2012; Mcwethy et al. 2013). However, by linking disturbance size to patch age diversity, the emergent results of our model suggest a mechanism for the creation of long-unburnt habitat patches, a phenomenon in flammable ecosystems that has perplexed managers and ecologists alike.

Whereas the results from the *Callitris* surveys and CA simulations provide a means by which to generalize the ecological effects of intentional burning on pyrodiversity at the local scale, the literature survey indicates this mechanism warrants consideration across a wide diversity of cultures and fire-prone ecosystems (Fig.4). Characterizing the human ‘footprint’ in these ecosystems has been challenging. Modern studies of indigenous burning, relegated to dwindling strongholds of indigenous culture (e.g., Yibarbuk et al. 2001; Laris 2002; Bliege Bird et al. 2008), are difficult to replicate and often disregarded as exceptional rather than typical. Remote sensing has provided insight into the effects of anthropogenic burning on ignition seasonality and increased habitat heterogeneity (Mbow et al. 2000; Laris 2002; Bowman et al. 2004; Petty and Bowman 2007; Bliege Bird et al. 2008), but the spatial resolution may still miss ecologically significant patterns and datasets are limited to decadal time spans.

Alternatively, paleoecological research appears equivocal as to the effects of humans on fire regime dynamics. Whereas some point to climate as the primary driver (Grisino-Mayer and Swetnam 2000; Marlon et al. 2008; Mooney et al. 2011), others have identified increases in fire occurrence that appear coeval with the appearance of pre-industrial human societies (Maxwell 2004; Fesenmyer and Christensen 2010; Pinter et al. 2011). Critical to this discrepancy, though largely overlooked, is the ecological evidence that human intervention (i.e., fire management) can significantly alter fire intensity and heterogeneity with little effect on the total extent of landscape burnt, which appears to be driven by climate (Gill et al. 2000; Van Wilgen et al. 2004; Archibald et al. 2009). Therefore, many paleoecological studies based on proxies of biomass burning, such as charcoal sediments, are likely dominated by climate-driven signals of landscape burning and possibly miss the spatial heterogeneity created by human activities.

Although our modeling results suggest how intentional burning can influence habitat heterogeneity and support fire-sensitive species like *Callitris*, understanding the specific outcomes of traditional fire management still depends on site-specific practices. Ecological knowledge of fire behavior and its outcomes enables people to decrease and increase fire size, among other fire regime characteristics, for specific purposes that may differ from the goals of contemporary management for conservation (Kimmerer and Lake 2001; Huffman 2013). All of the studies we surveyed cited multiple objectives of landscape burning (Fig.6), suggesting the practice is embedded in diverse production systems adapted to local climate, ecosystem processes, and disturbance regimes (Huffman 2013). However, the geographic extent of intentional burning derived from the literature survey indicates that the historical and contemporary effects of the human ‘footprint’ via fire management, and how this footprint has been altered (e.g., via fire suppression), warrant integration into broader, global models of pyrogeography (Fig.4, Krawchuk et al. 2009).

The extent to which the manipulation of natural resources for food and material culture is linked to ecological heterogeneity – and how changes in traditional systems of resource management are altering these relationships – has been widely discussed (Berkes et al. 2000). The diversity of objectives for intentional burning and frequency of citations in the literature (Fig.5) further corroborate, across six continents, prior ethnographic comparisons of traditional fire management systems (Stewart 1951; Lewis and Ferguson 1988; Kimmerer and Lake 2001). The frequency at which ‘cleaning’ landscapes (i.e., using fire to clear and maintain open vegetation) were mentioned (68%) attributes both the practicality of resource access and a positive esthetic value to landscapes altered by fire across multiple sites (Fig.5). The frequency of intentional burning being used to protect resources (49% among ethnographic studies) also suggests parallel goals between traditional fire management and contemporary prescribed burning programs used to mitigate wildfire risk.

It is naïve, however, to interpret the frequency of any objective as the degree to which it drives management practices. Many observed outcomes of intentional burning, such as patterns of habitat diversity or even the decreased prevalence of intense fires, may simply be unintended consequences of complex decision-making processes that incorporate multiple long- and short-term goals (Smith and Wishnie 2000). Thus, despite how well traditional fire management may support the goals of contemporary habitat conservation in terms of ecological outcomes, ‘recreating’ these ecological processes requires understanding the social landscapes in which these cultural practices emerged and have subsequently been altered by environmental and social change (Yibarbuk et al. 2001; Fowler 2013; McAdoo et al. 2013). The diversity of resources accessed and manipulated using fire (Figs.5 and 6) indicates that the successful coupling of humans and fire-prone ecosystems ultimately depends upon landscape-scale resource management. The ecological outcomes of traditional landscape burning have already shown promise for managing novel problems such as carbon sequestration, invasive species, and climate-induced increases in fire size (Murphy et al. 2009; Bliege Bird et al. 2012; McAdoo et al. 2013; Mcwethy et al. 2013). Equally compelling are the lessons and implications that these ‘emergent fire-adapted societies’ (Fowler 2013) potentially have for understanding contemporary human dimensions of fire management (e.g., Mason et al. 2012).

Taken together, the literature survey and our CA disturbance model show that contemporary land managers and conservationists ought to reconsider anthropogenic fire as part of the baseline processes shaping most of the world’s fire-prone ecosystems (Bowman et al. 2011). Of course, human decisions are embedded within a suite of interacting variables such as climate, substrate, and vegetation/fuel feedbacks that also shape fire regimes (Vigilante et al. 2004; Bond and Keeley 2005; Archibald et al. 2009). Yet the depth of traditional knowledge on the drivers of fire behavior (e.g., temperature, wind speed and direction, topography, fuel types and moisture) suggests clear intent and knowledge of fire management outcomes (Huffman 2013). The simulation results may be most applicable to spatially bound fire-prone habitats, such as the expanses of savanna where *Callitris* groves occur or the ‘yards’ and ‘corridors’ burned by indigenous people elsewhere (Lewis and Ferguson 1988). Also, the scaling effect described by our cellular automata can work in both directions—there are accounts of indigenous people intentionally setting large fires that do not fit the patch mosaic model (Kimmerer and Lake 2001).

## Conclusion

The obligate-seeding, fire-sensitive conifer *Callitris intratropica* requires long-unburnt habitat to persist in frequently burnt tropical savannas. The high density (1.1 ha^−1^) of *Callitris* groves surveyed in Kakadu National Park and central Arnhem Land reveals that, in these regions, long-unburnt patches commonly occur in the landscape, or did in the recent past. The consistently small grove sizes also suggest that long-unburnt habitat is the product of fine-scale patterns of indigenous burning (Laris 2002; Bowman et al. 2004; Vigilante et al. 2004; Bliege Bird et al. 2008). This interpretation is supported by the modeling outputs where, by increasing the density of discrete habitat patches, smaller disturbances increase the probability that landscapes contain a wider range of patch ages, even if median/mean patch age and fire frequency remain unchanged. Importantly, these temporal effects provide a possible mechanism by which anthropogenic patch burning could allow fire-sensitive plant species like *Callitris* to recruit, irrespective of overall fire frequency.

Our findings suggest that indigenous people created habitat mosaics as an emergent property of fires set for a variety of reasons. Modeling provides an important tool for understanding the effects of human-mediated disturbance and reconstructing mosaics that are often invisible in contemporary landscapes. However, in order to reestablish and adapt traditional systems, it is equally important to acknowledge and understand that the cultural processes and objectives driving these systems may differ dramatically from the goals of contemporary management and conservation. Although many practices have been lost or are in decline due to socio-ecological change, the widespread extent of traditional fire management (Fig.4) suggests there is a rich body of ecological and cultural knowledge that can provide insight to improve the management of fire-prone ecosystems. Clearly, social and cultural processes influence the future use of fire, so in this context, we can use traditional ecological knowledge to both inspire and frame field trials and modeling to guide management toward desired burning patterns and habitat composition (Wray and Anderson 2003; Storm and Shebitz 2006; Bilbao et al. 2010).

## Supporting Information

**Appendix S1** References on rural and indigenous landscape burning used in study.
