# Supplementary material for: Local and global pyrogeographic evidence that indigenous fire management creates pyrodiversity
Source: Ecol Evol. 2015 Apr 14;5(9):1908–18. doi: 10.1002/ece3.1494 (PMC4485971; doi:10.1002/ece3.1494)
Supplement: Supplementary file 1 [file ece30005-1908-sd1.docx]

Appendix S1. The research papers used to examine the objectives and geographic extent of rural and indigenous landscape burning organized by data type (direct ethnography, historical accounts, and descriptive accounts without reference to sources) and biome (‘mixed’ refers to multiple habitat types such as forest–savanna mosaics).

| **Data Type** | **Biome** | **References** |
| --- | --- | --- |
| Ethnographic | Arid savanna | Gould 1971, Kimber 1983, Bird et al. 2005, Burrows et al. 2006, Solomon et al. 2007, Vaarzon-Morel and Gabrys 2009 |
|  | Boreal forest | Gottesfeld 1994, Johnson 1999, Natcher et al. 2007, Miller 2010 |
|  | Subtropical forest | Coggins 2002 |
|  | Subtropical mixed | Johansson et al. 2012 |
|  | Subtropical savanna | Warren et al. 2001, Cassidy 2003, Bernard and Moetapele 2005, Kepe 2005, Sheuyange et al. 2005, Eriksen 2007, Angassa and Oba 2008, Butz 2009, Shaffer 2010 |
|  | Temperate forest | Barrett and Arno 1982, Chandler 1994, Anderson 2005, Lepofsky et al. 2005, Bustos-Schindler et al. 2010, Mason et al. 2012 |
|  | Temperate mix | Steward 1941, 1943, Stewart 1941, 1942, Lewis 1989, Boyd 1999, LaLande and Pullen 1999, Turner 1999, Ross 1999, Seijo 2005, Carroll et al. 2010, Coughlan 2013, Babai and Molnar 2014, Urgenson 2014 |
|  | Tropical forest | Hill and Baird 2003, Maxwell 2004 |
|  | Tropical mixed | Fairhead and Leach 1996, Russell-Smith et al. 1997, Mistry 1998, Kull 2002, Dampha et al. 2003, Lichang et al. 2003, King 2004, Dennis et al. 2005, McDaniel et al. 2005, Roveta 2008, Copsey et al. 2009, Huffman 2011, Rodriguez et al. 2011 |
|  | Tropical savanna | Thomson 1949, Lewis 1973, Jones 1980, O’Connell et al. 1983, Haynes 1985, Hough 1993, Russell-Smith et al. 1997, Mbow et al. 2000, Laris 2002, Mbata et al. 2002, Mistry et al. 2005, Rodriguez 2007, Russell-Smith et al. 2007, Hecht 2009, Walters 2010, Melo and Saito 2011, Fowler 2013, Welch et al. 2013 |
| Historical | Boreal Forest | Bele and Norderhaug 2013 |
|  | Temperate forest | Maxwell 1910, DeVivo 1991, Brose et al. 2001, Gammage 2008 |
|  | Temperate mixed | Stewart 1951, Day 1953, Hallam 1975, Norton 1979, Shinn 1980, Timbrook et al. 1982, Lewis and Ferguson 1988, Patterson III and Sassaman 1988, Hammett 1992, Brown 2000, Williams 2000, Condie and Raish 2002, Keeley 2002, Stewart 2002, Wry 2003, Gott 2005, Storm and Shebitz 2006, Williams 2009 |
|  | Tropical mixed | McEldowney 1979, Fensham 1997 |
|  | Tropical savanna | Preece 2013 |
| Descriptive | Temperate forest | Kurtulmuslu and Yazici 2003, Aagesen 2004 |
|  | Temperate mixed | Webb 1998, Métailié and Agnoletti 2006, Yallop et al. 2006, La Mantia et al. 2007, Shaoliang et al. 2007, Ascoli et al. 2009, Hartel 2013 |
|  | Tropical forest | Masipiqueña et al. 2000, Darlong 2002, Makarabhirom et al. 2002, London 2003 |
|  | Tropical mixed | Stott 1986, Bloesch 1999, Nanda and Sutar 2003, Schwartzmann et al. 2013 |
|  | Tropical savanna | Nacoulma et al. 2011 |

**REFERENCES**

Aagesen D. 2004. Burning monkey-puzzle: Native fire ecology and forest management in northern Patagonia. *Agr Hum Values* **21**:233-42.

Anderson MK. 2005. Tending the wild: Native American knowledge and the management of California's natural resources. Berkeley, CA: University of California Press.

Angassa A and Oba G. 2008. Herder perceptions on impacts of range enclosures, crop farming, fire ban and bush encroachment on the rangelands of Borana, Southern Ethiopia. *Hum Ecol* **36**:201-15.

Ascoli D, Beghin R, Ceccato R, *et* *al*. 2009. Developing an Adaptive Management approach to prescribed burning: a long-term heathland conservation experiment in north-west Italy. *Int J Wildland Fire* **18**:727–735.

Babai D and Molnar Z. 2014. Small-scale traditional management of highly species-rich grasslands in the Carpathians. *Agric Ecosyst Environ* **182**: 123–130.

Barrett SW and Arno SF. 1982. Indian fires as an ecological influence in the northern Rockies. *J For* **80**:647-51.

Bele, B and Norderhaug A. 2013. Traditional land use of the boreal forest landscape: Examples from Lierne, Nord-Trøndelag, Norway. *Norwegian J. Geogr.*, 67, 12–23.

Bernard, T and Moetapele, N. 2005. Desiccation of the Gomoti River: Biophysical process and indigenous resource management in Northern Botswana. *J Arid Environ* **63**: 256–283.

Bird DW, Bleige Bird R, and Parker CH. 2005. Aboriginal burning regimes and hunting strategies in Australia’s Western Desert. *Hum Ecol* **33**:443-64.

Bloesch U. 1999. Fire as a tool in the management of a savanna/dry forest reserve in Madagascar. *Applied Vegetation Science* **2**:117-24.

Boyd R. 1999. Strategies of Indian burning in the Willamette Valley. In Boyd R, (Ed.). Indians, fire and the land in the Pacific Northwest. Corvallis, OR: Oregon State University Press.

Brose P, Schuler T, Van Lear D*, et al.* 2001. Bringing fire back: the changing regimes of the Appalachian mixed-oak forests. *J For* **99**:30-35.

Brown H. 2000. Wildland burning by American Indians in Virginia. *Fire Management Today* **60**:29-39.

Burrows ND, Burbidge AA, Fuller PJ, *et al*. 2006. Evidence of altered fire regimes in the Western Desert region of Australia. *Conservation Science W Aus.* **5**:272-284.

Bustos-Schindler C, Le Quesne C, Gonzalez ME *et al*. 2010. Preliminary fire history and (multi) cultural practices in the Cachapoal river’s middle basin (34 degrees S), Central Chile. *Bosque* **31**:17–27.

Butz RJ. 2009. Traditional fire management: historical fire regimes and land use change in pastoral East Africa. *Int J Wildland Fire* **18**:442-50.

Carroll MS, Cohn PJ, Paveglio TB, *et al*. 2010. Fire Burners to Firefighters: The Nez Perce and Fire. *J For* **108**:71–76.

Cassidy L. 2003. Anthropogenic burning in the Okavango Panhandle of Botswana: Livelihoods and spatial dimensions. (PhD Dissertation). Gainesville, FL: University of Florida.

Chandler, P. 1994. Adaptive ecology of traditionally derived agroforestry in China. *Hum Ecol* **22**:415–442.

Coggins CR. 2002. Ferns and fire: village subsistence, landscape change, and nature conservation in China's southeast uplands. *J Cultural Geogr* **19**:12-159.

Condie CJ and Raish C. 2002. Indigenous and traditional use of fire in southwestern grassland, woodland, and forest ecosystems. St. Paul, MN: US Department of Agriculture, GTR-NC-231.

Copsey JA, Jones JPG, Andrianandrasana H, *et al*. 2009. Burning to fish: local explanations for wetland burning in Lac Alaotra, Madagascar. *Oryx* **43**:403–406.

Coughlan, MR. 2013. Errakina: pastora fire use and landscape memory in the Basque region of the French western Pyrenees. *J Ethnobiol* **33**:86–104.

Dampha A, Camara K, and Beck C. 2003. Management of forest fires through the involvement of local communities: The Gambia. Bangkok, Thailand: Food and Agriculture Organization of the United Nations, RAP 2003/8.

Darlong V. 2002. Traditional community-based fire management among the Mizo shifting cultivators of Mizoram in northeast India. Bangkok, Thailand: Food and Agriculture Organization of the United Nations, RAP 2002/25.

Day GM. 1953. The Indian as an ecological factor in the northeastern forest. *Ecology* **34**:329-46.

Deak B, Valko O, Toeroek P, *et* *al*. 2014. Grassland fires in Hungary – experiences of nature conservationists on the effects of fire on biodiversity. *Appl Ecol Environ Res* **12**:267–283.

de Melo MM and Saito CH. 2011. Regime de queima das caçadas com uso do fogo realizadas pelos Xavante no Cerrado. *Biodiversidade Brasileira*:97-109.

Dennis RA, Mayer J, Applegate G*, et al.* 2005. Fire, people and pixels: linking social science and remote sensing to understand underlying causes and impacts of fires in Indonesia. *Hum Ecol* **33**:465-504.

DeVivo MS. 1991. Indian use of fire and land clearance in the southern Appalachians. Asheville, NC: US Department of Agriculture, GTR-SE-69.

Dodgshon RA and Olsson GA 2006. Heather moorland in the Scottish Highlands: the history of a cultural landscape, 1600-1880. *J. Hist Geogr* **32**:21–37.

Eriksen C. 2007. Why do they burn the ‘bush’? Fire, rural livelihoods, and conservation in Zambia. *The Geographical Journal* **173**:242-56.

Fairhead J and Leach M. 1996. Enriching the landscape: social history and the management of transition ecology in the forest. savanna mosaic of the Republic of Guinea. *Africa* **66**:14-36.

Fensham R. 1997. Aboriginal fire regimes in Queensland, Australia: analysis of the explorers' record. *J Biogeogr* **24**:11-22.

Fowler, C. 2013. Ignition stories: indigenous fire ecology in the Indo-Australian monsoon zone. Durham, NC: Carolina Academic Press.

Fule PZ, Ramos-Gomez M, Cortes-Montano C, *et al*. 2011. Fire regime in a Mexican forest under indigenous resource management. *Ecol Appl* **21**:764–775.

Gammage B. 2008. Plain facts: Tasmania under aboriginal management. *Landscape Research* **33**:241-54.

Gott B. 2005. Aboriginal fire management in southeastern Australia: aims and frequency. *J Biogeogr* **32**:1203-08.

Gottesfeld LMJ. 1994. Aboriginal burning for vegetation management in northwest British Columbia. *Hum Ecol* **22**:171-88.

Gould RA. 1971. Uses and effects of fire among the Western Desert Aborigines of Australia. *Mankind* **8**:14-24.

Hallam SJ. 1975. Fire and hearth: a study of Aboriginal usage and European usurpation in south-western Australia: Australian Institute of Aboriginal Studies Canberra.

Hammett JE. 1992. The shapes of adaptation – historical ecology of anthropogenic landscapes in the southeastern United States. *Landsc Ecol* **7**:121–135.

Hartel T, Dorresteijn I, Klein C, *et al*. 2013. Wood-pastures in a traditional rural region of Eastern Europe: Characteristics, management and status. *Biol Conserv* **166**:267–275.

Haynes C. 1985. The pattern and ecology of munwag: traditional aboriginal fire regimes of north central Arnhem Land. *Proc Ecol Soc Aust* **13**:203-14.

Hecht SB. 2009. Kayapó savanna management: fire, soils, and forest islands in a threatened biome. In Woods WI, Teixeira WG, Lehmann J, Steiner C, WinklerPrins AMGA, and Rebellato L, (Eds). Amazonian dark earths: Wim Sombroek's vision. New York, NY: Springer.

Hill R and Baird A. 2003. Kuku—Yalanji rainforest Aboriginal people and carbohydrate resource management in the wet tropics of Queensland, Australia. *Hum Ecol* **31**:27-52.

Hough JL. 1993. Why burn the bush? Social approaches to bush-fire management in West African national parks. *Biol Conserv* **65**:23-28.

Huffman MR. 2011. Community-based fire management at La Sepultura Biopshere Reserve, Chiapas, Mexico. (PhD dissertation). Fort Collins, CO: Colorado State University.

Johansson MU, Fetene M, Malmer A*, et al.* 2012. Tending for cattle: traditional fire management in Ethiopian montane heathlands. *Ecol Soc* **17**:19.

Johnson LM. 1999. Aboriginal burning for vegetation management in northwest British Columbia. In Boyd R, (Ed.). Indians, fire and the land in the Pacific Northwest. Corvallis, OR: Oregon State University Press.

Jones R. 1980. Hunters in the Australian coastal savanna. In Harris DR, (Ed.). Human ecology in savanna environments. New York, NY: Academic Press.

Keeley JE. 2002. Native American impacts on fire regimes of the California coastal ranges. *J Biogeogr* **29**:303-20.

Kepe T. 2005. Grasslands ablaze: vegetation burning by rural people in Pondoland, South Africa. *South African Geographical Journal* **87**:10-17.

Kimber R. 1983. Black lightning: Aborigines and fire in central Australia and the Western Desert. *Arch in Oceania* **18**:38-45.

King, TG. 2004. A burning question? Fire, livelihoods and sustainability in the Navosa region of the Fiji Islands (PhD dissertation). Palmerston North, NZ: Massey University.

Kull CA. 2002. Madagascar's burning issue: the persistent conflict over fire. *Environment: Science and Policy for Sustainable Development* **44**:8-19.

Kurtulmuslu M and Yazici E. 2003. Management of forest fires through the involvement of local communities in Turkey. Bangkok, Thailand: Food and Agriculture Organization of the United Nations, RAP 2003/8.

LaLande J and Pullen R. 1999. Burning for a “fine and beautiful open country”: native uses of fire in southwestern Oregon. In Boyd R, (Ed.). Indians, fire and the land in the Pacific Northwest. Corvallis, OR: Oregon State University Press.

La Mantia T, Giaimi G, Veca DSLM, *et al.* 2007. The role of traditional *Erica arborea* L. management practices in maintaining northeastern Sicily’s cultural landscape. *For Ecol Manage* **249**:63–70.

Laris P. 2002. Burning the seasonal mosaic: Preventative burning strategies in the wooded savanna of southern Mali. *Hum Ecol* **30**:155-86.

Lepofsky D, Hallett D, Washbrook K, *et al.* 2005. Documenting pre-contact plant management on the northwest coast: an example of prescribed burning in the central and upper Fraser Valley, British Columbia. In Deur DE and Turner NJ (Eds). Keeping it living: traditions of plant use and cultivation on the northwest coast. Seattle: University of Washington Press.

Lewis HT. 1973. Patterns of Indian burning in California: ecology and ethnohistory. *Anthropological Papers* **1**.

Lewis HT. 1989. Ecological and technological knowledge of fire: Aborigines versus park rangers in northern Australia. *Am Anthropol* **91**:940-61.

Lewis HT and Ferguson TA. 1988. Yards, corridors, and mosaics: how to burn a boreal forest. *Hum Ecol* **16**:57-77.

Lichang Z, Long W, Yaqiao Z*, et al.* 2003. Community-based forest fire management in Wenyime village, Sanchahe township, Dayao county, Chuxiong Yi autonomous prefecture, Yunnan province, China. Bangkok, Thailand: Food and Agriculture Organization of the United Nations, RAP 2003/8.

Lightfoot KG and Parrish O. 2009. California Natural History Guides, Volume 96: California Indians and their environment: an introduction. University of California Press.

London S. 2003. Community-based fire management in Lao People’s Democratic Republic: past, present and future. Bangkok, Thailand: Food and Agriculture Organization of the United Nations, RAP 2003/8.

Makarabhirom P, Ganz D, and Onprom S. 2002. Community involvement in fire management: cases and recommendations for community-based fire management in Thailand. Bangkok, Thailand: Food and Agriculture Organization of the United Nations, RAP 2002/25.

Mason L, White G, Morishima G, *et al.* 2012. Listening and learning from traditional knowledge and western science: dialogue on contemporary challenges of forest health and wildfire. *J For* **110**:187–193.

Masipiqueña A, Persoon GA, and Snelder DJ. 2000. The use of fire in Northeastern Luzon (Philippines): conflicting views of local people, scientists and government officials. In Ellen R, Parkes P, and Bicker A, (Eds). Indigenous environmental knowledge and its transformations: critical anthropological perspectives. Amsterdam, The Netherlands: Harwood.

Maxwell AL. 2004. Fire regimes in north-eastern Cambodian monsoonal forests, with a 9300-year sediment charcoal record. *J Biogeogr* **31**:225-39.

Maxwell H. 1910. The use and abuse of forests by the Virginia Indians. William and Mary Quarterly Historical Magazine **19**:73-103.

Mbata KJ, Chidumayo EN and Lwatula CM. 2002. Traditional regulation of edible caterpillar exploitation in the Kopa area of Mpika district in northern Zambia. *J Insect Conserv* **6**:115–130.

Mbow C, Nielsen TT, and Rasmussen K. 2000. Savanna fires in east-central Senegal: distribution patterns, resource management and perceptions. *Hum Ecol* **28**:561-83.

McDaniel J, Kennard D, and Fuentes A. 2005. Smokey the Tapir: traditional fire knowledge and fire prevention campaigns in lowland Bolivia. *Soc Nat Resour* **18**:921-31.

McEldowney H. 1979. Archaeological and Historical Literature Search and Research Design, Lava Flow Control Study, Hilo, Hawaii. Honolulu, HI: US Army Engineer Division.

Métailié J and Agnoletti M. 2006. Mountain landscape, pastoral management and traditional practices in the northern Pyrenees (France). In Agnoletti M, (Ed.). The conservation of cultural landscapes. Oxfordshire: CABI.

Miller AM. 2010. Living with boreal forest fires: Anishinaabe perspectives on disturbance and collaborative forestry planning, Pikangikum First Nation, Northwestern Ontario. (PhD dissertation). Winnipeg, Canada: University of Manitoba.

Mistry J. 1998. Decision-making for fire use among farmers in savannas: an exploratory study in the Distrito Federal, central Brazil. *J Environ Manage* **54**:321-34.

Mistry J, Berardi A, Andrade V*, et al.* 2005. Indigenous fire management in the cerrado of Brazil: the case of the Kraho of Tocantins. *Hum Ecol* **33**:365-86.

Nacoulma BMI, Schumann K, Traore S*, et al.* 2011. Impacts of land-use on West African savanna vegetation: a comparison between protected and communal area in Burkina Faso. *Biodivers Conserv* **20**:3341–3362.

Nangendo G, Stein A, Gelens M*, et al.* 2002. Quantifying differences in biodiversity between a tropical forest area and a grassland area subject to traditional burning. *For Ecol Manage* **164**:109–120.

Nanda PK and Sutar PC. 2003. Management of forest fire through local communities: a study in the Bolangir, Deogarh and Sundergarh districts of Orissa, India. Bangkok, Thailand: Food and Agriculture Organization of the United Nations, RAP 2003/8.

Natcher DC, Calef M, Huntington O*, et al.* 2007. Factors contributing to the cultural and spatial variability of landscape burning by Native peoples of interior Alaska. *Ecol Soc* **12**:7.

Norton HH. 1979. The association between anthropogenic prairies and important food plants in western Washington. *Northwest Anthropological Research Notes* **13**:175-200.

O’Connell JF, Latz PK, and Barnett P. 1983. Traditional and modern plant use among the Alyawara of central Australia. *Econ Bot* **37**:80-109.

Patterson III WA and Sassaman KE. 1988. Indian fires in the prehistory of New England. In Nicholas GP, (Ed.). Holocene human ecology in northeastern North America. New York: Plenum.

Preece ND. 2013. Tangible evidence of historic Australian indigenous savanna management. *Austral Ecol*. 38:241-250.

Rodriguez I. 2007. Pemon perspectives of fire management in Canaima National Park, southeastern Venezuela. *Hum Ecol* **35**:331-43.

Rodriguez I, Albert P, La Rose C*, et al.* 2011. A study of the use of fire by Amerindian communities in South Rupununi, Guyana, with recommendations for sustainable land management. European Commission.

Ross JA. 1999. Proto-historical and historical Spokan prescribed burning and stewardship of resource areas. In Boyd R, (Ed.). Indians, fire and the land in the Pacific Northwest. Corvallis, OR: Oregon State University Press.

Roveta RJ. 2008. Traditional use of fire for the provision of ecosystem services: a case study in BRT Wildlife Sanctuary, India. (MSc thesis). Freiburg, Germany: University of Freiburg.

Russell-Smith J, Djoeroemana S, Maan J*, et al.* 2007. Rural livelihoods and burning practices in savanna landscapes of Nusa Tenggara Timur, Eastern Indonesia. *Hum Ecol* **35**:345-59.

Russell-Smith J, Lucas D, Gapindi M*, et al.* 1997. Aboriginal resource utilization and fire management practice in Western Arnhem Land, monsoonal northern Australia: notes for prehistory, lessons for the future. *Hum Ecol* **25**:159-95.

Sayles JS and Mulrennan, ME. 2010. Securing a Future: Cree Hunters’ Resistance and Flexibility to Environmental Changes, Wemindji, James Bay. *Ecol Soc* **15**:22

Seijo F. 2005. The politics of fire: Spanish forest policy and ritual resistance in Galicia, Spain. *Environmental Politics* **14**:380-402.

Schmerbeck J & Seeland K. 2007. Fire supported forest utilisation of a degraded dry forest as a means of sustainable local forest management in Tamil Nadu South India. *Land Use Policy* **24**:62–71.

Schwartzman S, Boas AV, Ono KY*, et al.* 2013. The natural and social history of the indigenous lands and protected areas corridor of the Xingu River basin. *Philos Trans R Soc B-Biological Sci* **368**.

Shaffer LJ. 2010. Indigenous fire use to manage savanna landscapes in southern Mozambique. *Fire Ecol* **6**:43-59.

Shaoliang Y, Ning W, Pang L, *et al.* 2007. Changes in livestock migration patterns in a Tibetan-style agropastoral system: A study in the Three-Parallel-Rivers Region of Yunnan, China. *Mt Res Dev* **27**:138–145.

Sheuyange A, Oba G, and Weladji RB. 2005. Effects of anthropogenic fire history on savanna vegetation in northeastern Namibia. *J Environ Manage* **75**:189-98.

Shinn DA. 1980. Historical perspectives on range burning in the inland Pacific Northwest. J Range Mgmt **33**:415-123.

Solomon TB, Snyman HA and Smit GN. 2007. Cattle-rangeland management practices and perceptions of pastoralists towards rangeland degradation in the Borana zone of southern Ethiopia. *J Environ Manage* **82**:481–494.

Steward JH. 1941. Culture element distributions: XIII Nevada Shoshoni. *Anthropol Rec* **4**:209–359.

Steward JH. 1943. Culture element distributions: XXXIII Northern and Gosiute Shoshoni. *Anthropol Rec* **8**:263–392.

Stewart OC. 1941. Culture element distributions: XIV Northern Paiute. *Anthropol Rec* **4**:361–446.

Stewart OC. 1942. Culture element distributions: XVIII Ute-Southern Paiute. *Anthropol Rec* **6**:231–360.

Stewart OC. 1951. Burning and natural vegetation in the United States. *Geographical Review* **41**:317-20.

Stewart OC. 2002. Forgotten Fires. Edited with introductions by Henry T. Lewis and M. Kat Anderson. Norman: University of Oklahoma Press.

Storm L and Shebitz D. 2006. Evaluating the purpose, extent, and ecological restoration applications of indigenous burning practices in southwestern Washington. *Ecol Restor* **24**:257.

Stott P. 1986. The spatial pattern of dry season fires in the savanna forests of Thailand. *J Biogeogr*:345-58.

Thomas E, Douterlungne D, Vandebroek I, *et al*. 2011. Human impact on wild firewood species in the Rural Andes community of Apillapampa, Bolivia. *Environ Monit Assess* **178**:333–347.

Thomson DF. 1949. Arnhem Land: explorations among an unknown people. Part II. The people of Blue Mud Bay. *The Geographic Journal* **113**:1-8.

Timbrook J, Johnson JR, and Earle DD. 1982. Vegetation burning by the Chumash. *Journal of California and Great Basin Anthropology* **4**:163-86.

Turner NJ. 1999. “Time to burn”: traditional resource use of fire to enhance resource production by Aboriginal peoples in British Columbia. In Boyd R, (Ed.). Indians, fire and the land in the Pacific Northwest. Corvallis, OR: Oregon State University Press.

Urgenson L, Schmidt AH, Combs J, *et al*. 2014. Traditional Livelihoods, Conservation and Meadow Ecology in Jiuzhaigou National Park, Sichuan, China. *Hum Ecol* **42**:481–491.

Vaarzon-Morel P and Gabrys K. 2009. Fire on the horizon: contemporary Aboriginal burning issues in the Tanami Desert, central Australia. *GeoJournal* **74**:465-76.

Walters GM. 2010. The Land Chief’s embers: ethnobotany of Batéké fire regimes, savanna vegetation and resource use in Gabon. (PhD dissertation). London, UK: University College London.

Warren A, Kosonen J, Katundu M*, et al.* 2001. Causes and consequences of anthropogenic fire in Mulanje Mountain Forest Reserve, southern Malawi. (MSc Thesis). Canterbury, UK: University of Kent.

Webb N. 1998. The traditional management of European heathlands. *J Appl Ecol* **35**:987-90.

Welch JR, Santos RV, Flowers NM *et* *al*. 2013. Na primeira margem do rio: território e ecologia do povo Xavante de Wedezé. Rio de Janeiro: Museo do Índio-FUNAI.

Williams E. 2009. Maori fire use and landscape changes in southern New Zealand. *Journal of the Polynesian Society* **118**:175-89.

Williams GW. 2000. Early fire use in Oregon. *Fire Management Today* **60**:13-20.

Wry J, Anderson M. 2003. Restoring Indian-set fires to prairie ecosystems on the Olympic Peninsula. *Ecological Restoration* **21**:296-301.

Yallop AR, Thacker JI, Thomas G, *et* *al*. 2006. The extent and intensity of management burning in the English uplands. *J Appl Ecol* **43**: 1138–1148.
